# Malaria elimination practices in China from the perspective of health system and social development

**DOI:** 10.1186/s40249-025-01406-5

**Published:** 2026-01-08

**Authors:** Xinyi Song, Zuokun Liu, Na Li, Long Chen, Mengze Liu, Jiajun Liu, Yuyang Zhang, Minmin Wang, Minghui Ren

**Affiliations:** 1 Department of Global Health, School of Public Health, Peking University, Beijing, China; 2 Beijing Institute for Health Development, Peking University, Beijing, China; 3 China Center for Health Development Studies, Peking University, Beijing, China; 4 Institute for Global Health, Peking University, Beijing, China

**Keywords:** Malaria elimination, China, Health system, Social development

## Abstract

**Background:**

China, once a malaria-endemic country, has developed a comprehensive set of extensive strategies and accumulated practical experience over 70 years of malaria elimination efforts. On June 30, 2021, China was officially certified by the World Health Organization as malaria-free. Substantial research has already summarized China’s malaria control experience from a technical standpoint. This study aims to examine China’s malaria elimination practices from a new perspective of the health system and social development.

**Methods:**

Semi-structured interviews were conducted with key informants, including national malaria program managers, renowned scholars, and technical personnels from China, international organizations, and high-burden countries in Africa. Interviews were conducted from July 2023 to July 2025, and data were analyzed using the thematic framework method.

**Results:**

A total of 42 participants responded to the interview, and 7 key components from social development was proposed. The thematic analysis identified key factors influencing the achievement of malaria elimination in China. Specifically, 57.14% of experts mentioned agricultural crop types, 66.67% highlighted health education, 64.29% noted the working environment, 52.38% referred to employment opportunities, 59.52% addressed water and sanitation, 71.43% emphasized the living environment, and all experts underscored the critical role of the health system.

**Conclusions:**

Social development is closely intertwined with malaria elimination, as advancements in healthcare, infrastructure, and community engagement are essential for ensuring the long-term success of malaria control efforts. Future studies could further explore the impact of specific factors on malaria elimination, thereby contributing valuable insights to global malaria elimination efforts.

**Graphical Abstract:**

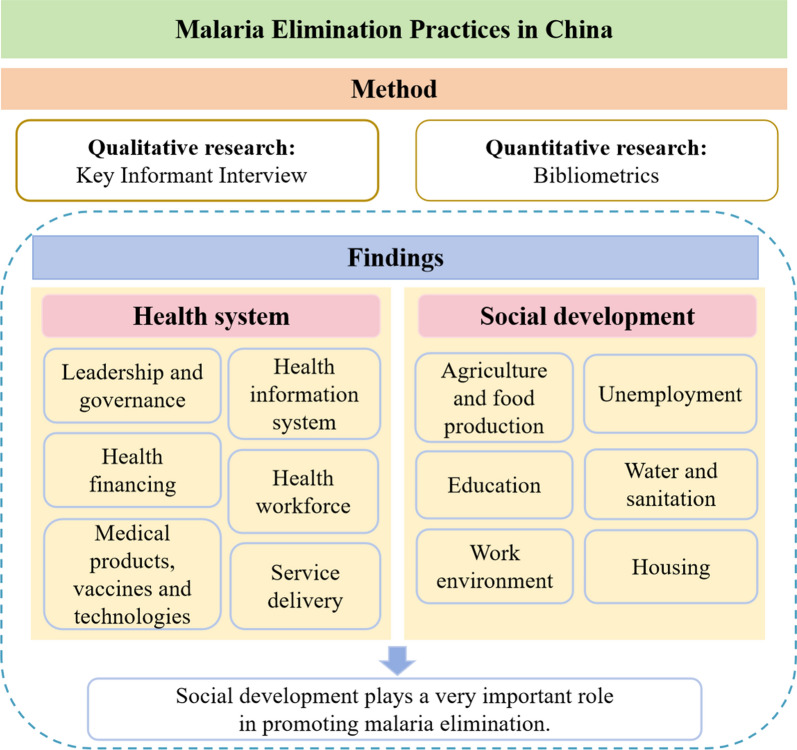

**Supplementary Information:**

The online version contains supplementary material available at 10.1186/s40249-025-01406-5.

## Background

Malaria remains a major public health problem worldwide. Ending the epidemic of malaria is one of the key targets of the health and wellbeing goal in the Sustainable Development Goals (SDGs). Global elimination of malaria faces considerable challenges, with the disease imposing a substantial burden worldwide, particularly in sub-Saharan Africa. According to the *World Malaria Report 2024* released by World Health Organization (WHO), there were an estimated 263 million malaria cases globally in 2023, resulting in approximately 597,000 deaths [1].

Since the 1950s, China has made remarkable progress and significant breakthroughs in malaria control and elimination. Following an on-site assessment by the WHO, China was officially certified malaria-free on June 30, 2021 [2]. Transitioning from a malaria-endemic country to a malaria-free nation, China has not only effectively controlled the epidemic but also accumulated substantial practical experience, which has had an important impact on the development of its public health system and has contributed to advancing the global malaria elimination agenda [3].

Many studies on China’s successful efforts to eliminate malaria tend to focus primarily on the technical and scientific aspects of the country’s approach. These include the development and deployment of effective anti-malarial drugs, the use of insecticide-treated nets, and innovations in diagnostic tools and vector control methods. The bibliometric analysis of China’s malaria experience highlights a focus on the 1-3-7 norm [4], the development of anti-malarial products such as artemisinin and insecticide-treated nets, as well as research on emerging drug resistance (Supplemental Materials). However, these studies often overlook the equally critical contributions of broader social development to malaria control and elimination.

**Fig. 1:**
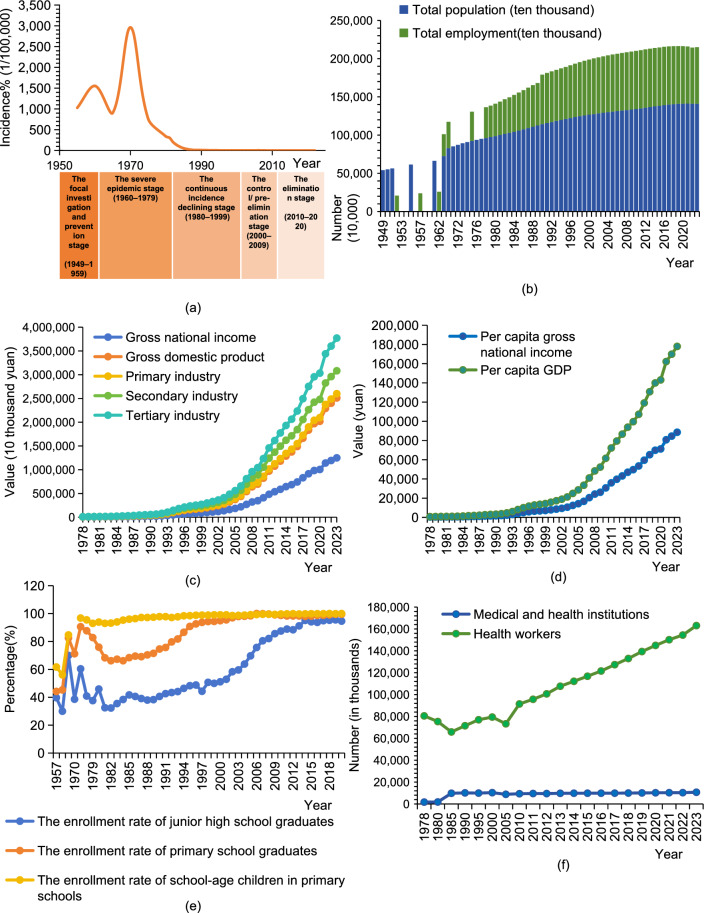
Changes in the incidence of malaria and related indicators of social development and the health system in China, 1949–2020. **a** Reported incidence of indigenous malaria. **b** Total population and number of employed persons. **c** Economic indicators including gross national income, gross domestic product and the total values of the three major industries. **d** Per capita national income and per capita gross domestic product. **e** The enrollment rates of junior high school students, primary school students and school-age children. **f** The number of medical and health institutions and health workers

WHO has published a series of documents, including the *Global Technical Strategy for Malaria 2016–2030* and the *Global Framework for Responding to Malaria in Urban Areas*, highlighting the critical role of social development in malaria control. Furthermore, multiple studies have emphasized the role of social development in achieving malaria elimination. For instance, a 2013 study published in *The Lancet* suggested that socio-economic development may serve as a highly effective and sustainable malaria intervention in the long term [5]. Another study demonstrated that rapid urban development in Hainan Province, China, significantly accelerated malaria elimination [6]. Additionally, based on data from the *China Health Statistical Yearbook* and *the China Statistical Yearbook*, a correlation between the incidence of malaria and China’s socioeconomic, demographic, educational, and health system indicators was identified from 1949 to 2020 (Fig. 1).

Therefore, this study constructs a theoretical framework and identifies the key dimensions of China’s malaria control practices from the perspectives of social development and health system. The findings are intended to provide control recommendations for high-burden countries of malaria and to promote China’s deeper involvement in global malaria elimination.

## Methods

**Fig. 2:**
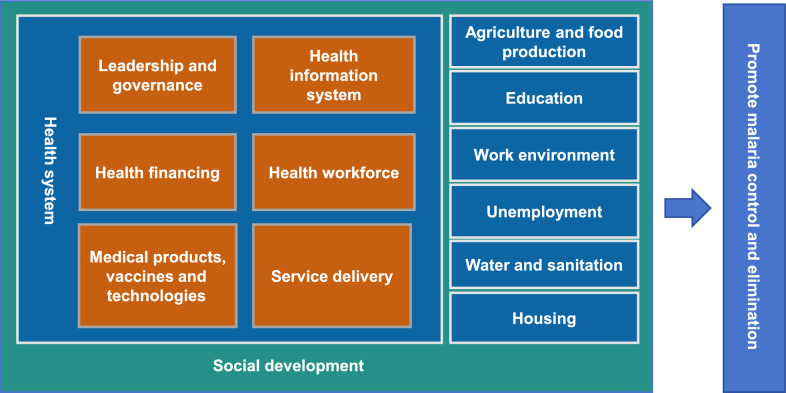
Theoretical framework of malaria control and elimination practices in China based on the perspective of health system and social development

Based on the WHO’s health system framework of six building blocks and the rainbow model of health determinants [7], we constructed a theoretical framework of the malaria control from the perspectives of the health system and social development (Fig. 2). This framework served as the outline of the qualitative interview guide to organize and analyze the practices of malaria prevention and control.

### Respondents

Individuals engaged in malaria control and elimination efforts were considered key informants in this study, particularly those from China and Africa. They were categorized into three groups: (1) officials, academics, or technical personnels from health related departments in China; (2) counterparts from international organizations; and (3) counterparts from malaria-endemic countries in Africa.

### Sampling strategy

A key informant interview was conducted among participants of the China-Africa Malaria Elimination Cooperation Seminar hosted by the Peking University Institute for Global Health and the “Belt and Road” Africa Aid Training Program organized by the Jiangsu Institute of Parasitic Diseases. In October 2016, the Jiangsu Institute of Parasitic Diseases was designated as WHO Collaborating Centre for Malaria Elimination Research and Training, serving as a global platform for professional training, academic discussions, and experience exchange in malaria elimination. The China-Africa Malaria Elimination Cooperation Seminar extended invitations based on the expert list from the Malaria Policy Advisory Group (MPAG). Experts from China, international organizations, and high malaria burden African countries were selected through purposive sampling. In total, 42 respondents from 17 countries were included (Table 1). The experts from the United States and Spain were affiliated with international organizations and possessed extensive expertise in malaria control.

**Table 1:** Country distribution, type and number of respondents

Classification	Subtype	Number
Country	Benin	1
	Burkina Faso	3
	Cameroon	3
	China*	13
	Comoros	1
	Democratic Republic of the Congo	1
	Gabon	1
	The Gambia	3
	Namibia	2
	Nigeria	3
	Solomon Islands	2
	South Africa	1
	South Sudan	2
	Spain*	1
	Tunisia	2
	Uganda	2
	United States of America*	1
Working Institute	Health related departments in government	25
	Research Institute/University	7
	Hospitals/health facilities	10

^*^A Chinese expert served in the WHO; A Spanish expert served in the WHO; An American expert served in a university

### Data collection

Semi-structured interviews were conducted using an interview guide developed based on the theoretical framework (Supplemental Material). The main content of the interview focused on how China, from the perspective of the health system and social development, has unique malaria control practices that can make positive contributions to the global efforts to eliminate malaria. All interviewers underwent standardized training prior to conducting the interviews. The interviews were conducted in Chinese, English, or French and continued until data saturation was achieved. With the participants’ informed consent, all interviews were audio-recorded for the ease of transcription.

### Data analysis

The interview content was transcribed verbatim into Microsoft Word 2021*(Microsoft Corporation, Redmond, WA, USA)* documents. Data were analyzed through the thematic framework analysis. Through multiple discussions within the research team, themes and sub-themes were identified (Table 2). Based on the thematic framework, the interview materials were coded, and key information was extracted to draw conclusions.

**Table 2:** Thematic framework

Theme	Sub-theme
1. China's malaria elimination practices from the health system perspective	1.1 Leadership and governance
	1.2 Health information system
	1.3 Health financing
	1.4 Health workforce
	1.5 Medical products, vaccines and technologies
	1.6 Service delivery
2. China's malaria elimination practices from the social development perspective	2.1 Agriculture and food production
	2.2 Education
	2.3 Work environment
	2.4 Unemployment
	2.5 Water and sanitation
	2.6 Housing

### Quality control

Interviews were conducted by two or more trained interviewers who recorded and transcribed the discussions in real time. For data analysis, two authors were responsible for the transcription, while a third author conducted cross-verification to ensure accuracy.

## Results

### Key informant interview

A total of 42 interviewees were included, comprising 30 males and 12 females, aged 30–60 years. Among the respondents, 25 were from health-related departments, 7 from research institutions or universities, and 10 from hospitals or other medical institutions (Table 1). From the perspective of social development, experts highlighted the impact of the following factors on malaria control and elimination: 57.14% mentioned agriculture, 66.67% mentioned the health education, 64.29% mentioned the work environment, 52.38% mentioned employment opportunities, 59.52% mentioned water and sanitation, and 71.43% mentioned living environment. Almost all experts emphasized the importance of the health system for malaria control and elimination.

### Malaria elimination practices in China

#### Agricultural crop types

Agricultural factors—including crop types, farming environments, food safety, and development levels—directly or indirectly influence malaria transmission.

Respondents from China stated that *“Crop substitution, such as replacing rice cultivation with cash crops, along with changes in land use patterns, can reduce mosquito breeding and biting, thereby controlling infection sources and interrupting transmission routes”* (officer in health ministry)*.*

China has extensive rice paddies, which serve as breeding grounds for mosquito larvae. The promotion of rice-fish co-culture systems has significantly reduced mosquito density in fields and achieved remarkable effects in malaria elimination, while also offering advantages such as low investment, higher rice yields, and considerable economic returns. Therefore, agriculture constitutes an indispensable component of malaria elimination efforts.

#### Health education

The educational attainment and overall literacy have served as a foundation for the effective implementation of malaria control and elimination efforts. With social development, China’s education level has improved significantly, leading to enhanced population health literacy and substantial improvements in personal hygiene habits. China has consistently emphasized health education and awareness campaigns related to disease prevention, enabling residents to gradually acquire awareness and knowledge of epidemic control.

According to respondents of China, *“As education levels have risen, so has public health awareness… Courses on infectious disease control and elimination have been introduced in school curricula, helping students systematically learn and master knowledge related to disease prevention and treatment”* (researcher in the university).

Multiple Chinese experts mentioned that in the past, residents did not proactively use anti-malarial products such as mosquito nets to prevent bites. However, with increased public health awareness, more people have come to understand the necessity of malaria control, actively sought treatment at professional institutions, and familiarized themselves with information on therapeutic drugs. Several African officials pointed out that people in Africa do not voluntarily use insecticide-treated nets or even repurpose them for fishing, highlighting the need for further educational efforts.

#### Working environment

China’s rapid socio-economic development has been accompanied by continuous improvements in infrastructure, leading to significant enhancements in working conditions for its people. Initiatives such as the National Sanitary City Campaign and the New Rural Development Program have driven remarkable environmental transformations, effectively altering mosquito breeding habitats and curbing the transmission of malaria.

As a respondent from an international organization noted, *“China is undergoing rapid urbanization, with notable social progress, well-developed infrastructure, and improved living conditions. These advancements play a crucial role in reducing the burden of malaria”* (officer in health ministry)*.*

In contrast, health officials and technical experts from African countries highlighted the relative weakness of infrastructure and challenging working environments across the continent, which pose obstacles to effective malaria control efforts.

#### Employment opportunities

Since the implementation of China’s reform and opening-up policy, Chinese society has developed rapidly. Compared to rural areas, cities provide more employment opportunities and higher resident incomes, enabling residents to obtain better living and working conditions as well as higher-quality medical services.

A Chinese respondent emphasized, *“Better employment and development prospects in cities enable people to move away from high-risk malaria areas, which contributes to malaria control efforts”* (officer in health ministry).

A Nigerian respondent noted, *"With increased income from work, people can access better healthcare, educational resources, and living conditions, all of which facilitate malaria control and elimination"*(officer in health ministry).

#### Water and sanitation

The quality and safety of drinking water, the upgrading of water conservancy infrastructure, and the construction of roads and drainage systems are key factors in malaria control efforts.

A Chinese respondent pointed out, *“The Chinese government prioritized improving water sources, leading to widespread access to clean drinking water and flush toilets, while ponds around residential areas were eliminated—and malaria disappeared accordingly”* (officer in health ministry).

A Gambian respondent mentioned, *“In my view, ensuring drinking water quality is one of the most critical factors influencing malaria control and elimination”* (officer in health ministry).

#### Living environment

Housing conditions significantly impact malaria control by influencing vector-borne transmission. Multiple interviewees indicated a correlation between housing design, wall materials, and the prevalence of malaria among residents, noting that dwellings with wooden or bamboo walls are associated with higher infection rates.

A Chinese respondent remarked, *“The construction of housing is crucial… From thatched huts in the 1970s to stone-slab houses in the 1980s, and now multi-story buildings, malaria cases have declined accordingly”* (officer in health ministry).

Another Chinese respondent added, *“With socio-economic development, improved housing conditions—such as the installation of air conditioning and window screens—have reduced human-mosquito contact, leading to a decline in infection rates. This represents the most direct change”* (researcher in the university).

However, several African health officials pointed out that the common practice of sleeping outdoors in many African communities increases human-mosquito contact, posing a challenge to malaria control efforts.

#### Health system

##### Leadership

Experts widely agree that the robust health system is crucial for malaria control and elimination. The most distinctive feature and central factor in China’s success is the top-down leadership and high-level commitment from the Chinese government. China’s vertical management system, which operates without profit motives, combined with strong administrative capacity and multi-sectoral participation, enables timely, efficient, and sustainable policy implementation.

A respondent from China said, *“China has established a disease control system that operates from the grassroots health stations to county-level reporting and national verification. It is a top-down process involving coordinated actions at all levels… China’s disease control system is well-structured, and the execution power of personnel at all levels is strong. This network is highly effective”* (officer in health ministry).

##### Health information system

The Chinese government has consistently prioritized the development of health information systems, transitioning from paper-based reporting to a direct online reporting system. Continuous improvements have been made in malaria vector surveillance and drug resistance monitoring systems, leading to the establishment of the 1-3-7 norm [4], which ensures effective outbreak control. Several African health officials noted that most African regions had not yet established digital surveillance systems, making direct application of the 1-3-7 norm challenging [4]. They expressed hope for enhanced China-Africa Cooperation in digital health infrastructure in the future.

##### Sustained funding

Most respondents mentioned that the Chinese government has consistently allocated substantial funding to malaria elimination, supporting scientific research, personnel training, and system strengthening. After the suspension of Global Fund projects, the central government continued to provide substantial funding for malaria control.

##### Health workforce development

Experts also agree that health workforce capacity building and specialist team development are the cornerstones of malaria elimination. Through long-term training and practical experience, China has cultivated a workforce proficient in both theory and field operations.

##### Medical products, vaccines and technologies

China’s contributions in malaria control products and technologies are particularly noteworthy. The artemisinin discovered by Professor Tu Youyou and her colleagues revolutionized malaria treatment.

Respondent of China said, *“China, as the origin of artemisinin, has developed all artemisinin-based products on the market today”* (researcher in the university).

The use of insecticide-treated nets for malaria control was also an innovation pioneered in China.

##### Specialized health service delivery

China’s approach to malaria control and elimination has always adhered to the principle of “adapting to local conditions and providing differentiated guidance”. Historically, China relied primarily on a three-tier health network and the Patriotic Health Campaign for malaria control, mobilizing “barefoot doctors” to disseminate health knowledge. Today, a joint control and elimination mechanism has been established to consolidate elimination achievements across provinces, continuously strengthening the health service system and enhancing service delivery capacity.

## Discussion

This study analyzed China’s malaria control and elimination strategies from both a health system and social development perspective, highlighting the significant role of broader health determinants in achieving malaria elimination.

Overall, the expertise recognized that social development has been crucial to China’s malaria control and elimination, including the key factors of agricultural crop types, health education, working environment, employment opportunity, water and sanitation, and living environment [8–10]. This framework reflects the combined effect of the health system and social development on malaria control strategies. This study found that previous studies also remain predominantly focused on summarizing technical aspects, such as the development of antimalarial drugs and the “1-3-7” norm [4, 11, 12]. Through expert interviews, it was determined that various domains of social development—including agriculture, education, the working environment, unemployment, water and sanitation, and housing—played crucial roles in the malaria elimination process and should receive greater emphasis in the future. Consequently, this study streamlined the description of technical elements like the health system, placing stronger emphasis on the significant contributions of diverse social development factors to malaria elimination. This approach aims to provide malaria-affected countries with novel insights and strategies for controlling and eliminating the disease.

Specifically, socio-economic development can support malaria control efforts by improving living conditions and enhancing the sustainability of effective interventions, thereby contributing to the reduction of malaria incidence in China. Stagnant water around residences and water-related development projects, such as large dams, can create vector breeding sites and increase the risk of malaria infection. However, since 1980, improved water and sanitation facilities have been a national priority in China’s rural development programs. China’s “Twelfth Five-Year Plan” emphasized the importance of safe drinking water, adequate sanitation, and hygiene [13]. Ponds and rice paddies serve as breeding grounds for malaria vector mosquitoes [14]. In 1956, the national goal of malaria elimination was integrated into China’s agricultural and development strategies. In 1982, agricultural departments in regions such as Guangzhou dredged ditches and cleaned water supply networks to reduce mosquito breeding sites and habitats, thereby minimizing human-vector contact [8]. Socio-economic and housing factors also play significant roles in malaria transmission. For instance, open eaves, high population density, poor working conditions, and low household income can increase the risk of malaria infection. Since the 1980s, China has made a series of published policies focusing on improving education levels to enhance public knowledge of malaria control. Examples include disseminating scientific information on malaria control through media campaigns, displaying banners in public places, strengthening health education for border-crossing populations, and incorporating malaria control into primary and secondary school curricula [15].

This study proposed a novel framework for China’s malaria elimination practices, which not only encompasses various aspects of the health system but also seeks to extract unique or successful malaria control strategies from the perspective of social development, thereby offering a Chinese approach to global malaria elimination efforts. The health system and social development are, in fact, mutually reinforcing [4]. China has not only prioritized establishing a comprehensive health system but has also implemented poverty alleviation programs that significantly improved housing, living conditions, and communication networks. Malaria control efforts should not be confined to the health sector alone, as sectors like agriculture, education, and water resources management have also played crucial roles. Therefore, this study recommends that future development cooperation projects related to malaria control be integrated with infrastructure construction initiatives and involve multi-sectoral collaboration to formulate comprehensive malaria prevention plans. In the long term, social development plays an exceptionally important role in promoting malaria control, underscoring its significant research relevance.

This study interviewed officials, experts, scholars, and technical personnels from health sectors in China, international organizations, and high malaria burden African countries, which may enhance the reliability of the findings. However, a limitation of this study is that the interviewees were confined to experts within the health system domain. Malaria control additionally involves fields such as environment, agriculture, water resources, economy, demographics, and diplomacy, requiring valuable inputs from experts in other disciplines. Therefore, as future research progresses, it is essential to invite scholars and professionals from a broader range of fields to participate in interviews, to further explore the specific roles played by various dimensions of social development in malaria control and elimination.

## Conclusions

China’s malaria elimination effort is not only a public health achievement but also a demonstration of how systematic governance can drive social progress. Social development is closely linked to malaria elimination. This study identified seven key components of malaria control and elimination through expert interviews. We anticipate that future research will further explore China’s malaria control practices from a social development perspective, thereby providing evidence for enhanced global health collaboration.

## Supplementary Information

Supplementary material 1.

## Data Availability

Data for analysis was extracted from publications. The codes used for our analyses are available upon request from the corresponding author RM.

## Abbreviations

SDGs: Sustainable Development Goals
WHO: World Health Organization
