# Supplementary material for: Malaria elimination practices in China from the perspective of health system and social development
Source: Infect Dis Poverty. 2026 Jan 8;15:3. doi: 10.1186/s40249-025-01406-5 (PMC12781699; doi:10.1186/s40249-025-01406-5)
Supplement: Supplementary file 1 — Supplementary material 1. [file 40249_2025_1406_MOESM1_ESM.docx]

**Malaria Elimination Practices in China from the Perspective of Health system and Social Development**

Xinyi Song^1^, Zuokun Liu^1^, Na Li^1^, Long Chen^1^, Mengze Liu^1^, Jiajun Liu^1^, Yuyang Zhang^1^, Minmin Wang^1,2^^,3*^, Minghui Ren^1,2,3,4*^

1 Department of Global Health, School of Public Health, Peking University, Beijing, China

2 Beijing Institute for Health Development, Peking University, Beijing, China

3 China Center for Health Development Studies, Peking University, Beijing, China.

4 Institute for Global Health, Peking University, Beijing, China

**Appendix A:**

1. Interview guide

1.1 Interview questions for Chinese experts

1. Based on your work and research, could you briefly describe the process through which China achieved malaria elimination?
2. For each stage you mentioned, how did the establishment and reform of China’s healthcare system contribute to malaria control, including monitoring of imported cases, case management, and elimination efforts?
3. From the perspective of the healthcare system, what are the unique characteristics and experiences of China's malaria control efforts, including aspects like health policies, financing, monitoring, human resources, primary healthcare, cross-departmental collaboration, and involvement of the social and private sectors? What factors have been central to these efforts, and which practices should be considered for replication in Africa?
4. From a broader societal development perspective, how do you understand and recognize the role of social development in China’s malaria elimination efforts from the founding of the country to the reform and opening up period? Which non-health-related policies, measures, regulations, or actions have positively or negatively influenced malaria control in China? Are there any typical cases?
5. In recent years, China has made significant socio-economic progress, with rapid urbanization, advancements in agriculture, industry, and manufacturing, and extensive infrastructure development. In this context, what factors have been the core drivers of malaria elimination? Are there any adverse factors? What policies and measures did China adopt to achieve the global goal of malaria elimination?
6. In addition to the healthcare system and social development factors, what other successful experiences in China’s malaria elimination efforts are worth further exploration and summary, and how have they contributed to global malaria elimination?

1.2 Interview questions for international experts

1. China has achieved malaria elimination in 2021. What do you perceive as the key experiences or successful strategies in malaria elimination within the country? What unique characteristics and insights can be identified from the perspective of technical implementation and health system?
2. In recent years, China has experienced rapid urbanization, characterized by significant social development, infrastructural enhancements, and improvements in living conditions. What social development factors do you believe have been pivotal in the process of malaria elimination in China?
3. Several developing countries, including Cabo Verde, Belize, Azerbaijan, and Tajikistan, have successfully achieved malaria elimination in recent years. What distinct practices in China stand out as valuable lessons compared to these countries?
4. From the perspective of health systems and social development, what positive contributions does China's experience in successfully eliminating malaria offer to global malaria elimination efforts?

1.3 Interview questions for African experts

(A) Personal Background

1. Which country do you come from? Which institution are you affiliated with? What is your position and how many years of experience do you have? What is your educational and training background? Have you been involved in malaria control work?
2. Is this your first time participating in a Chinese malaria training program? Have you attended similar programs in your country?
3. Have you been invited to attend similar training programs in other countries, such as the US, France, UK, WHO, Global Fund, or RBM (Roll Back Malaria)?

(B) Application of China’s Malaria Control Experience in African Countries

1. Could you briefly describe the malaria situation in your country?
2. When your country develops malaria control measures, does it consider China’s experience in malaria control? What do you think are China’s successful practices or approaches in malaria elimination?
3. In recent years, several African and developing countries have achieved malaria elimination, such as Cabo Verde, Belize, Azerbaijan, and Tajikistan. Compared to these countries, which Chinese practices are of greater interest to you and more worthy of adaptation?
4. Regarding strengthening the health system's capacity to combat malaria (including health financing, monitoring and data reporting systems, human resources, primary healthcare systems, cross-departmental collaboration, and private sector involvement), what are the main strategies in your country? Have any Chinese practices (including policies, technologies, theories, or products) been applied? If so, what are they?
5. Have the agricultural or water resources sectors in your country considered the requirements for malaria elimination?
6. What are your country’s main strategies for malaria elimination in other areas? Have any Chinese experiences (including policies, technologies, theories, or products) been applied? If so, what are they?
7. How do you assess the current application of China’s experience in your country? What are the key factors influencing its implementation?

2. Bibliometrics:

2.1 Method

This study uses CNKI and the Web of Science Core Collection as the literature retrieval databases. With the assistance of VosViewer1.6.20 (*Centre for Science and Technology Studies, Leiden University, The Netherlands*) and Microsoft Excel2021(*Microsoft Corporation, Redmond, WA, USA*), the retrieved literature was analyzed for research trends and keywords. We include Chinese and English literature related to China's malaria prevention, control, and elimination experiences from the perspectives of health systems and social development, published from the inception of the self-built database to December 31, 2024.

**In this study, the core databases of CNKI and web of science were used as literature retrieval databases, and only "article" and "review article" were reserved in the core database of web of science.** The search formula is: ((((((((((TS=(leadership*)) OR TS=(governance*)) OR TS=(information system*)) OR TS=(financing*)) OR TS=(workforce*)) OR TS=(medical product*)) OR TS=(vaccine*)) OR TS=(technologies*)) OR TS=(service*))AND TS=(China*)) AND TS=(malaria*) AND ((((TS=(prevention*)) OR TS=(control*)) OR TS=(elimination*)))；((((((((TS=( agriculture*)) OR TS=(food production*)) OR TS=(education*)) OR TS=(work environment*)) OR TS=(unemployment*)) OR TS=(water and sanitation*)) OR TS=(housing*)) AND TS=(China*)) AND TS=(malaria*) AND ((((TS=(prevention*)) OR TS=(control*)) OR TS=(elimination*)))。

The inclusion criteria for the literature are: (1) The theme matches the search keywords; (2) The main research content is to examine China's malaria prevention and control practices from the perspective of health systems or social development. The exclusion criteria for the literature are: non-peer-reviewed publications such as conference proceedings, reports, newspapers, news articles, etc.; and literature that is not accessible.

In addition, this study utilized the statistical analysis function of the Web of Science Core Collection database to obtain results such as annual publication count and keywords. VOS viewer 1.6.20 was employed to create a co-occurrence map of the keywords. The parameters for generating the keyword co-occurrence map were set as follows: all keywords were selected as the unit of analysis, with a minimum frequency threshold of three for keywords from CNKI and three for keywords from the Web of Science Core Collection.

2.2 Results

In the Web of Science Core Collection, 151 articles discussed China’s malaria elimination practices from a health system perspective, while an additional 61 articles addressed it from a social development perspective (Figure 1). In the China National Knowledge Infrastructure (CNKI), 315 articles focused on malaria elimination from a health-system perspective and 20 from a social-development perspective (Figure 2).


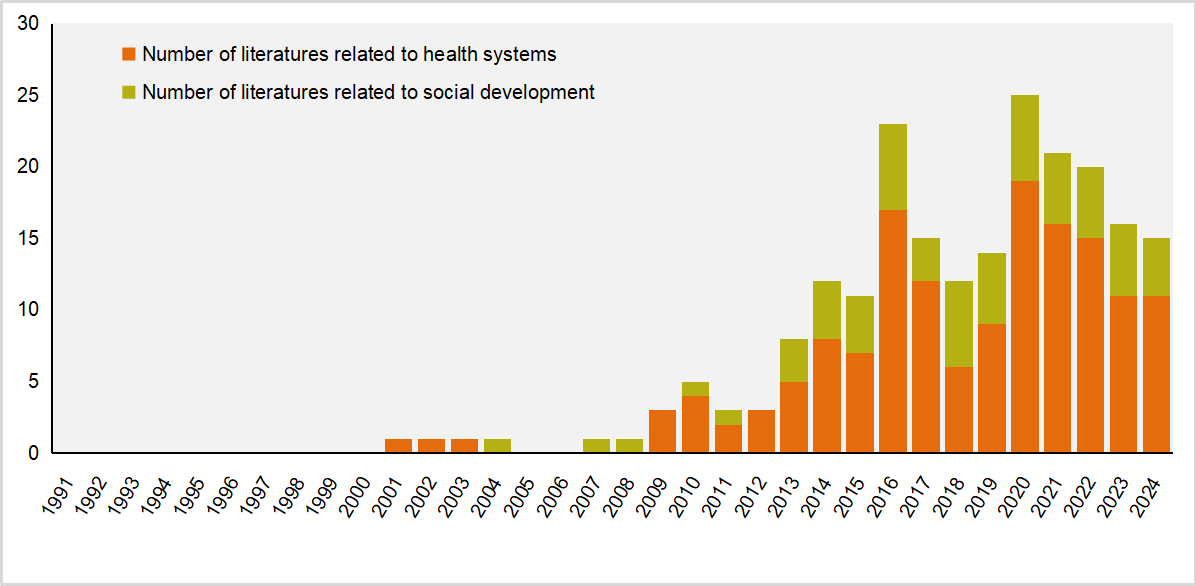


**Fig. 1** Publication year of web of science core collection literature


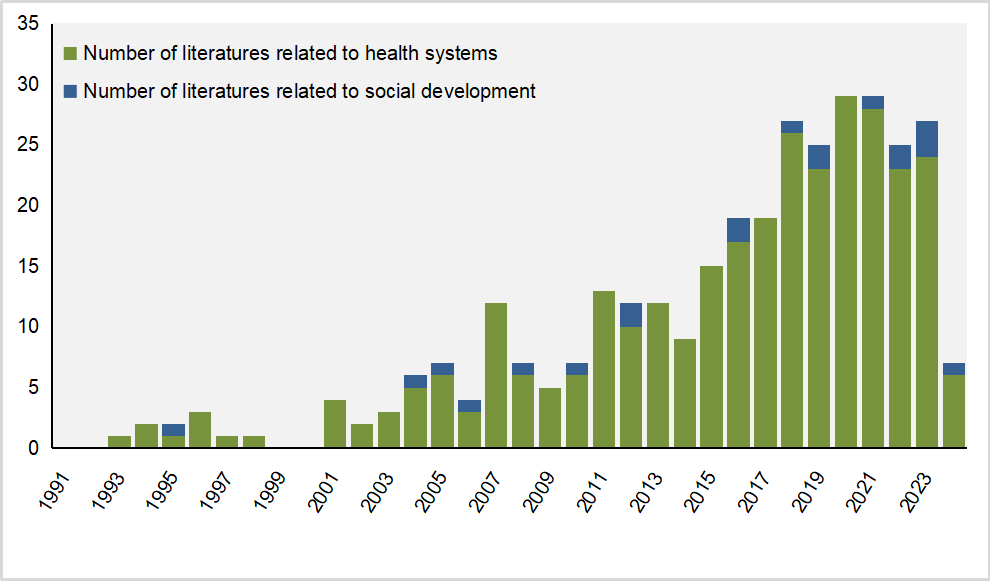


**Fig. 2** Publication year of CNKI literature

Related publications emerged after 2000, and gradually increased after 2010, and reached a peak around 2020, shortly before China received WHO malaria elimination certification in 2021. More than 75% of the total articles focused on health system. However, both Chinese and English articles on China’s malaria prevention and control practices from the perspective of social development have been consistently scarce, indicating that there is currently limited research in this area.

After merging synonyms, the keyword co-occurrence of the included literature is shown in Figures 4 and 5. In the included English literature examining China’s malaria control and elimination practices from the perspective of health system, 54 nodes that appeared 3 times or more formed a keyword co-occurrence map with “malaria elimination” as the core (Figure 3). The most frequently occurring keywords include surveillance, malaria transmission, and imported malaria. In the included Chinese literature, 39 nodes that appeared 3 times or more formed a co-occurrence map centered on with “malaria” as the core (Figure 4). The most frequently occurring keywords include elimination, imported malaria, and artemisinin. Due to the limited number of articles examining China’s malaria control practices from the perspective of social development, it was not possible to create a keyword co-occurrence map for this category. This suggests that current research hotspots are concentrated at the technical research level, with many research outputs summarizing China’s malaria prevention and elimination experiences, anti-malaria strategies, and applications, while there is little research addressing social development issues.


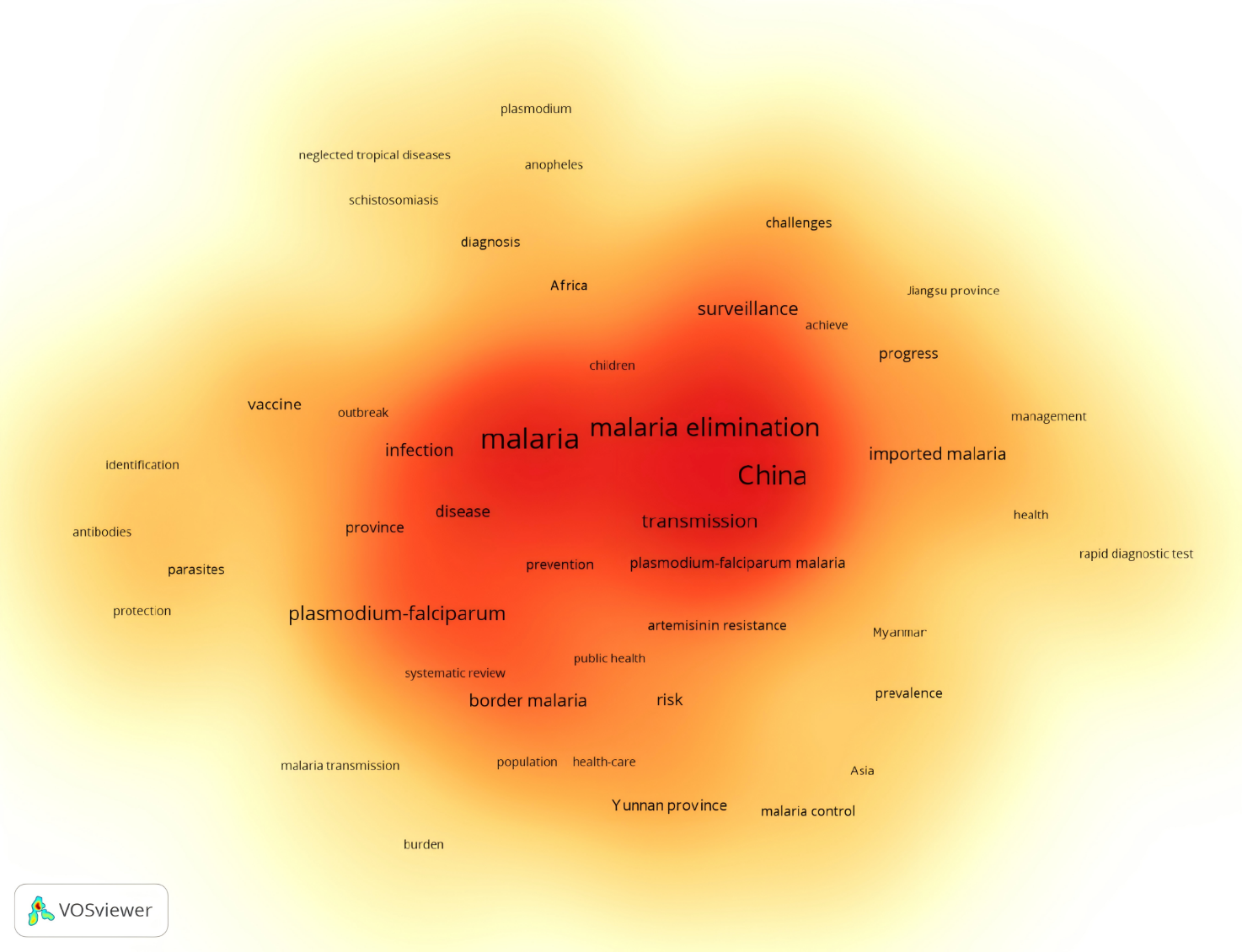


**Fig. 3** Co-occurrence of keywords in English literature on malaria control in China


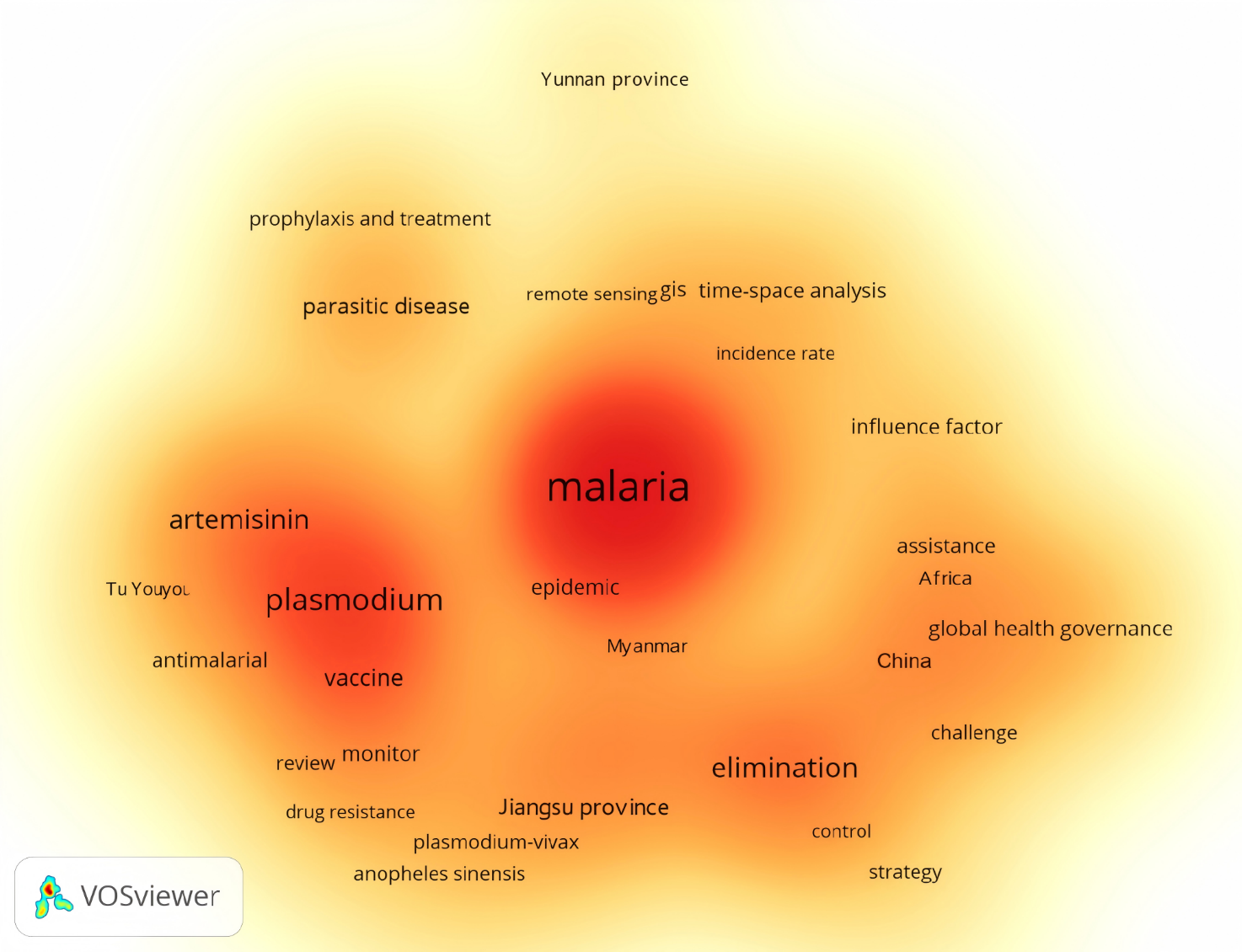


**Fig. 4** Co-occurrence of keywords in Chinese literature on malaria control in China
